# Chronic Helminth Infection Does Not Exacerbate *Mycobacterium tuberculosis* Infection

**DOI:** 10.1371/journal.pntd.0001970

**Published:** 2012-12-20

**Authors:** Marc P. Hübner, Kristin E. Killoran, Michael Rajnik, Samuel Wilson, Kevin C. Yim, Marina N. Torrero, Christopher P. Morris, Boris Nikonenko, Jorge C. G. Blanco, Val G. Hemming, Edward Mitre

**Affiliations:** 1 Department of Microbiology and Immunology, Uniformed Services University of the Health Sciences, Bethesda, Maryland, United States of America; 2 Institute for Medical Microbiology, Immunology and Parasitology, University Hospital Bonn, Bonn, Germany; 3 Department of Pediatrics, Uniformed Services University of the Health Sciences, Bethesda, Maryland, United States of America; 4 Virion Systems, Inc., Rockville, Maryland, United States of America; 5 Sigmovir Biosystems Inc., Rockville, Maryland, United States of America; 6 Sequella, Inc., Rockville, Maryland, United States of America; University of Edinburgh, United Kingdom

## Abstract

**Background:**

Chronic helminth infections induce a Th2 immune shift and establish an immunoregulatory milieu. As both of these responses can suppress Th1 immunity, which is necessary for control of *Mycobacterium tuberculosis* (MTB) infection, we hypothesized that chronic helminth infections may exacerbate the course of MTB.

**Methodology/Principal Findings:**

Co-infection studies were conducted in cotton rats as they are the natural host for the filarial nematode *Litomosoides sigmodontis* and are an excellent model for human MTB. Immunogical responses, histological studies, and quantitative mycobacterial cultures were assessed two months after MTB challenge in cotton rats with and without chronic *L. sigmodontis* infection. Spleen cell proliferation and interferon gamma production in response to purified protein derivative were similar between co-infected and MTB-only infected animals. In contrast to our hypothesis, MTB loads and occurrence and size of lung granulomas were not increased in co-infected animals.

**Conclusions/Significance:**

These findings suggest that chronic filaria infections do not exacerbate MTB infection in the cotton rat model. While these results suggest that filaria eradication programs may not facilitate MTB control, they indicate that it may be possible to develop worm-derived therapies for autoimmune diseases that do not substantially increase the risk for infections.

## Author Summary

Tuberculosis prevalence is high in areas that are endemic for helminths, suggesting that many people are chronically infected with both pathogens. As parasitic helminths can suppress the host immune system to facilitate their own survival, they frequently impact the host immune response to bystander antigens. Thus, while helminth infections ameliorate allergies and autoimmune diseases, they also decrease immune responses elicited by vaccines. Several studies have shown that helminth exposure impairs *Mycobacterium tuberculosis*-specific immune responses, raising the possibility that helminth infections may decrease the host's ability to control *M. tuberculosis* infection. To test this, we analyzed whether chronic infection of cotton rats with the filarial worm *Litomosoides sigmodontis* exacerbates the course of *M. tuberculosis* infection. Cotton rats are an excellent model organism to study human *M. tuberculosis* as they develop, in contrast to mice, distinct granuloma formation during infection. In addition, cotton rats are the natural host for *L. sigmodontis*, a nematode that establishes long-lived infections (>2 years) with circulating microfilariae in these animals. The results of this study demonstrate that chronic filarial infection does not exacerbate *M. tuberculosis-*associated pathology or mycobacterial burdens in cotton rats and suggest that filaria-induced immunoregulation can be overcome to respond effectively to newly acquired infections.

## Introduction

Tuberculosis and helminth infections affect approximately one third of the world's population. The geographic distributions of both diseases overlap substantially, making co-infections with both pathogens common.

In contrast to infections with most bacterial, viral, protozoan, and fungal pathogens, chronic helminth infections are associated with Th2 immune responses characterized by eosinophilia, elevated IgE levels, and production of type 2 cytokines such as IL-4, IL-5, and IL-13 [1]. Over time, however, chronic helminth infections induce immunoregulatory networks through regulatory T cells, alternatively activated macrophages, and the inhibitory cytokines IL-10 and TGFβ [1]. The effects of these immune responses on the host are complex. While helminth-induced immunoregulation enhances parasite survival in the host, it also impacts the immune response to bystander antigens. As a benefit to the host, helminth-induced immunoregulation appears to play a role in protection against allergies and autoimmune diseases [2], [3], . Negatively, though, helminth infections hamper the development of adequate immune responses to vaccines like BCG, tetanus toxin, and cholera vaccine [8], [9], [10].

As infections with *Mycobacterium tuberculosis* (MTB) require a protective IFNγ-driven Th1 immune response [11], and as both Th2 and immune regulatory responses induced by helminths can suppress Th1 immunity, it has been hypothesized that helminth infections may impair development of a protective immune response against MTB [12], [13]. To date, however, the clinical impact chronic helminth infections have on co-infections with organisms such as MTB, *Plasmodium*, or HIV is controversial and not sufficiently understood [13].

The primary limitation of experimental helminth and mycobacteria co-infection studies reported to date is the utilization of mouse models of mycobacterial infection. In humans, mycobacterial infections routinely result in the development of distinct granulomas with central caseating necrosis. The formation of these granulomas in humans is believed to be necessary for immunologic control of the bacteria. Murine mycobacterial infections, however, develop diffuse infection patterns without well-formed granulomas. Recently, this limitation in rodent models of mycobacteria has been overcome with the development of the cotton rat model of MTB infection. In this system, granulomas consist of macrophages that surround the bacteria and exhibit central caseous necrosis similar to human granulomas [14]. In addition to being a useful model for mycobacterial disease, cotton rats are the natural host for the long-lived filarial nematode *Litomosoides sigmodontis* [15]. The adults of this parasite live in the pleural space and, after 7–8 weeks, release their offspring, the microfilariae, which circulate in the blood.

For our experiments, cotton rats chronically infected with *L. sigmodontis* and uninfected controls were challenged intranasally with MTB and nine weeks later euthanized to evaluate PPD-specific splenocyte proliferation and IFNγ production, lung histology, and bacterial load in the lung and spleen by quantitative culture.

## Methods

### Animals and infection protocols

Experiments were performed with 6–8-week-old female cotton rats (*Sigmodon hispidus*) that were obtained from Virion Systems, Inc. and maintained at the Uniformed Services University of the Health Sciences (USU) animal facility. The cotton rats are considered inbred since they have been brother sister mated in excess of 20 generations. Animals were housed individually and obtained water and food ad libitum.

Cotton rats were infected by subcutaneous injection with 100 infectious *L. sigmodontis* L3 larvae in media (RPMI-1640, Mediatech) as previously described [16]. After the development of a chronic *L. sigmodontis* infection at 11 weeks post infection, a subset of helminth-infected animals and uninfected controls were challenged with 5×10^4^ *M. tuberculosis* bacteria (H37Rv) by intranasal inoculation.

The *M. tuberculosis* strain H37Rv was originally obtained from the Institute Pasteur, Paris, France (a kind gift of Prof. G. Marchal) and is now maintained at Sequella, Inc.. *Mycobacteria* stock was prepared by suspending *Mycobacteria* in 7H9 broth supplemented with bovine serum albumin (BSA), dextrose, and catalase. The mycobacterial suspension was cultured two successive times in roller bottles at 37°C for 7 days. The final culture was washed in PBS with 0.05% Tween 80, resuspended in PBS with 0.01% BSA and 0.05% Tween 80, aliquoted, and frozen at −80°C. CFU of the frozen aliquots were determined after thawing by plating serial 10-fold dilutions on 7H10 agar.

### Ethics statement

Animal experiments were performed under a protocol approved by the USU Institutional Animal Care and Use Committee.

### Determination of worm burden

*L. sigmodontis* worms reside in the pleural cavity where they molt into adult worms around 30 days post infection [15]. Around 8 weeks post infection, microfilariae, the offspring of adult worms, are released and enter the peripheral blood. Peripheral blood microfilaria counts were performed as described previously [16] at eleven weeks (immediately before MTB challenge) and at the end of the study, twenty weeks post *L. sigmodontis* infection. In brief, 10 µl of peripheral blood was obtained and added to 1 ml of ACK lysis buffer (Quality Biological, Inc.). After centrifugation the supernatant was removed and the remaining pellet was completely analyzed for microfilariae numbers by microscopy. Those numbers were divided by 10 to obtain microfilariae per µl of peripheral blood. As the microfilarial burden was too high to count at 20 weeks post *L. sigmodontis* infection, we resuspended the microfilaria-containing pellet in 100 µl PBS and evaluated microfilaria levels in 10 µl of this suspension to obtain total numbers of microfilariae per µl of peripheral blood. Adult worms were enumerated 20 weeks after helminth infection by careful removal from the pleural cavity using a dissection probe.

### Automated differential cell blood count

Peripheral blood was obtained by orbital bleeding or puncturing the inferior vena cava following laparotomy under a lethal dose of sodium pentobarbital from 5-week and 11-week *L. sigmodontis* infected cotton rats and uninfected controls. Automated differential cell blood counts were performed using a Bayer Advia 120 differential leukocyte counter.

### Spleen cell proliferation and IFNγ production

At different time points after *L. sigmodontis* infection (5, 11, 20 weeks post infection) cotton rats were euthanized and spleen cells were isolated. Single cell suspensions were obtained (0.22 µm filter, BD Bioscience) and red blood cells lysed (ACK lysis buffer). Spleen cell proliferation and IFNγ production was determined from 2×10^6^/ml spleen cells cultured with 20 µg/ml *M. tuberculosis* Tuberculin PPD (Statens Serum Institut), 20 µg/ml crude *L. sigmodontis* adult worm antigen (LsAg, prepared as previously described [17]), 10 µg/ml *Staphylococcus* enterotoxin B (SEB, Toxin Technology, Inc.), or cell culture media alone (Iscove's modified Dulbecco's media (Mediatech), 10% FCS (Valley Biomedical), 1% L-glutamine (Mediatech), 1% insulin-transferrin-selenium (Invitrogen Inc.), 1% penicillin-streptomycin (Mediatech)). After 48 h BrdU was added for 16 h and cellular proliferation subsequently determined according to the manufacturer's recommendations (Roche Diagnostics GmbH). In parallel cultures, IFNγ production was determined in cell culture supernatants after 72 h using a cotton rat specific ELISA according to the manufacturer's recommendations (R&D Systems, Inc.).

### Assessment of MTB infection

For microscopic evaluation of histopathology, the left lung was inflated through the trachea to its normal volume with 10% buffered formalin and sections were stained with hematoxylin and eosin (H&E). Modified acid-fast tissue stain was used to confirm the presence of acid-fast bacilli. The area of the lung containing granulomas was estimated in a blinded fashion by a single investigator (VGH).

Colony-forming units (CFUs) were assessed from equal amounts of homogenized tissue of spleen and the right lung and plated in 10-fold serial dilutions on 7H10 agar.

### Statistical analysis

Statistical analysis was performed using GraphPad Prism software (GraphPad Software). Differences between multiple groups were tested for significance using the Kruskal-Wallis test followed by Dunn's post-hoc multiple comparisons. Differences between two groups were tested for significance with the Mann-Whitney-U-test. Correlations were tested using the spearman test. Impact of chronic *L. sigmodontis* infection on MTB was investigated in two independent experiments. Results are shown as representative examples from one experiment.

## Results

### Chronic *L. sigmodontis* infection induces eosinophilia and a hyporesponsive mileu

**Figure 1:**
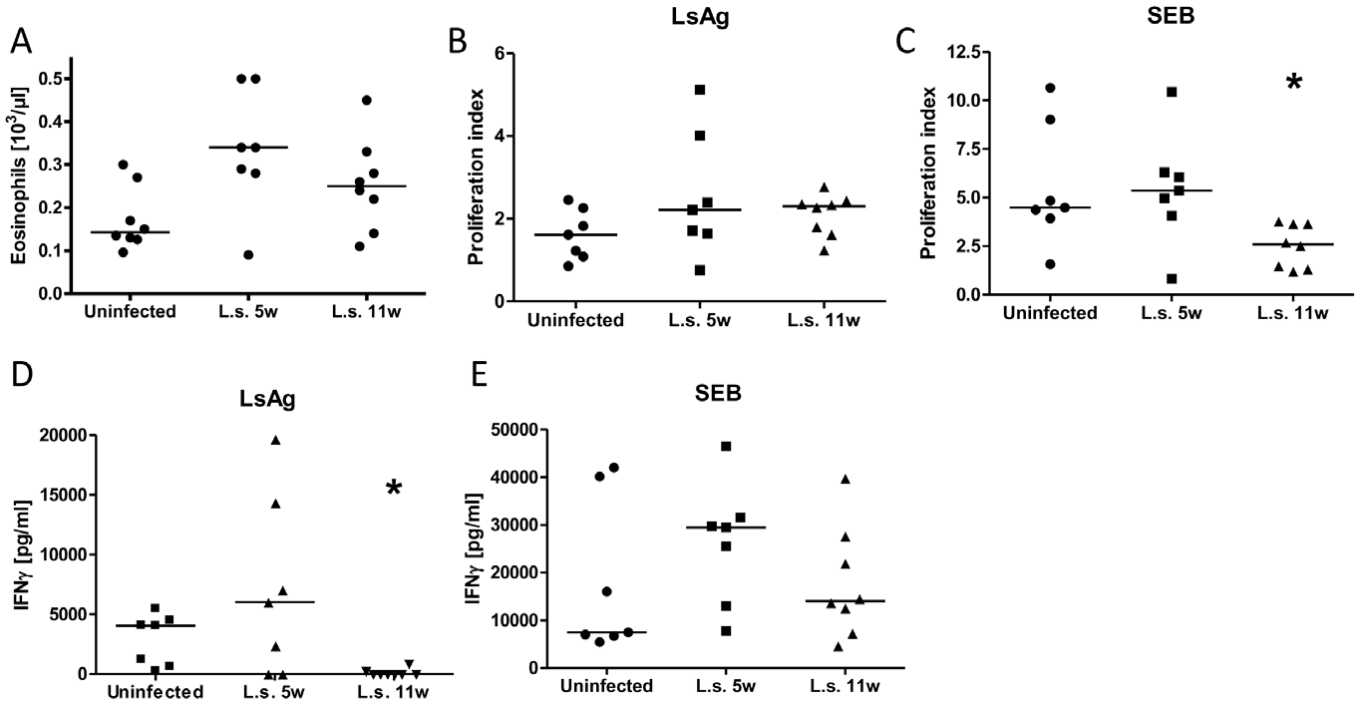
Chronic *L. sigmodontis* infection induces eosinophilia and a hyporesponsive milieu. *A*, peripheral blood eosinophil counts from uninfected, 5, and 11 week-*Litomosoides sigmodontis* (L.s.) infected cotton rats. In vitro spleen cell proliferation (*B*, *C*, as proliferation index (OD of stimulated cells/baseline)) and IFNγ production (*D*, *E*) in response to *L. sigmodontis* antigen (LsAg) or *Staphylococcal* enterotoxin B (SEB) from cotton rats that were either uninfected or infected with *L. sigmodontis* for 5 or 11 weeks. Statistical significance between groups was analyzed by the Kruskal-Wallis test, followed by Dunn's post-hoc multiple comparisons. Single stars show significant differences compared to uninfected animals. *p<0.05.

To confirm that *L. sigmodontis* infection induces a Type 2 immune response and a hyporesponsive milieu in cotton rats, we infected cotton rats with 100 L3 larvae and analyzed eosinophil counts, spleen cell proliferation, and IFNγ production 5 and 11 weeks after infection. Those time points were chosen as they reflect acute infection, with adult worms present in the pleural cavity prior to the release of microfilariae, and chronic infection after onset of microfilaria release into the circulation. As seen in figure 1A, *L. sigmodontis* infection of cotton rats induces a substantial increase in numbers of peripheral eosinophils at both 5 and 11 weeks post infection, though the differences between these timepoints and uninfected cotton rats did not reach statistical significance.

While *L. sigmodontis* antigen induced non-specific proliferation of splenocytes from uninfected cotton rats, a trend towards increased proliferation in response to parasite antigen was observed in splenocytes from infected animals (Fig. 1B). Splenocytes from 11 week, but not 5 week, *L. sigmodontis* infected cotton rats exhibited significantly reduced proliferation in response to stimulation with SEB, a superantigen which induces polyclonal activation of T-cells, compared to uninfected controls (Fig. 1C).

Parasite-specific IFNγ production from splenocytes was reduced in animals that were chronically infected for 11 weeks with *L. sigmodontis* compared to uninfected and 5 week-infected animals (Fig. 1D). While splenocytes from 5 week-infected cotton rats produced more IFNγ in response to SEB than uninfected animals, by 11 weeks of infection IFNγ production from splenocytes had decreased compared to 5 weeks (Fig. 1E). These findings, in addition to the decreased proliferation induced by SEB at 11 weeks, are consistent with the development of an immune regulated state during chronic filariasis.

### Co-infection of cotton rats with *L. sigmodontis* and *M. tuberculosis*

**Figure 2:**
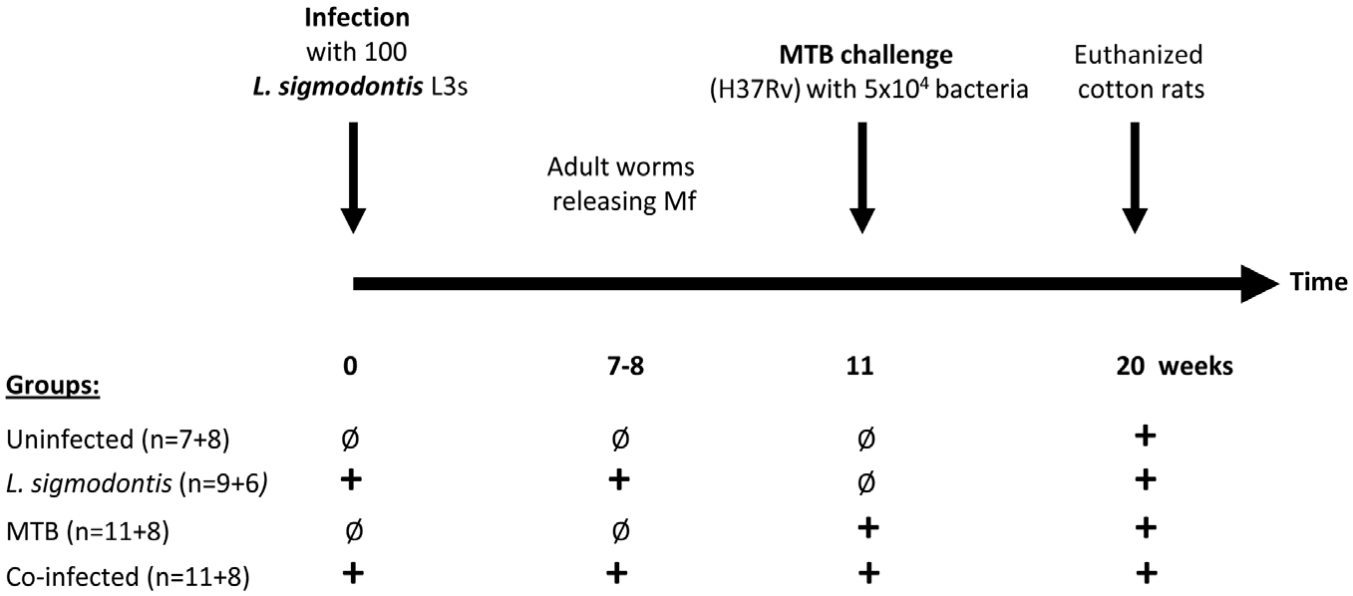
Experimental setup. Cotton rats were infected with 100 infectious *Litomosoides sigmodontis* L3 larvae and intranasally challenged with 5×10^4^ *M. tuberculosis* (MTB) bacteria 11 weeks later, a timepoint by which they had developed a patent (microfilaria (Mf)-releasing) filaria infection. At 20 weeks cotton rats were euthanized and immunological and histological studies performed. Numbers in brackets indicate the number of analyzed cotton rats in the first and second experiment.

We tested whether chronic helminth infection exacerbates MTB infection by infecting cotton rats with 100 *L. sigmodontis* L3 larvae. Subsets of these animals and uninfected controls were challenged 11 weeks later by intranasal inoculation of 5×10^4^ *M. tuberculosis* bacteria and euthanized 9 weeks later (Fig. 2). This experiment was conducted twice.

During the first co-infection experiment, four out of 24 cotton rats that were infected with *L. sigmodontis* died before the MTB challenge. Two additional cotton rats that received MTB-only challenge died 3 and 5 days post MTB inoculation. No animals died at later timepoints and none of the co-infected animals died during the experiment. In the second experiment two out of eight *L. sigmodontis-*only infected cotton rats (18 weeks post *L. sigmodontis* infection), but none of the MTB only or co-infected cotton rats, died post *L. sigmodontis* infection. We assume that the observed death of cotton rats was due to the duration of the experiments rather than as a consequence of excessive filarial or MTB burden. These deceased cotton rats were not included in the analysis. As no co-infected animals died, inclusions of these animals in the final analysis would have only strengthened our ultimate conclusion that chronic helminth infection does not hinder control of MTB.

**Figure 3:**
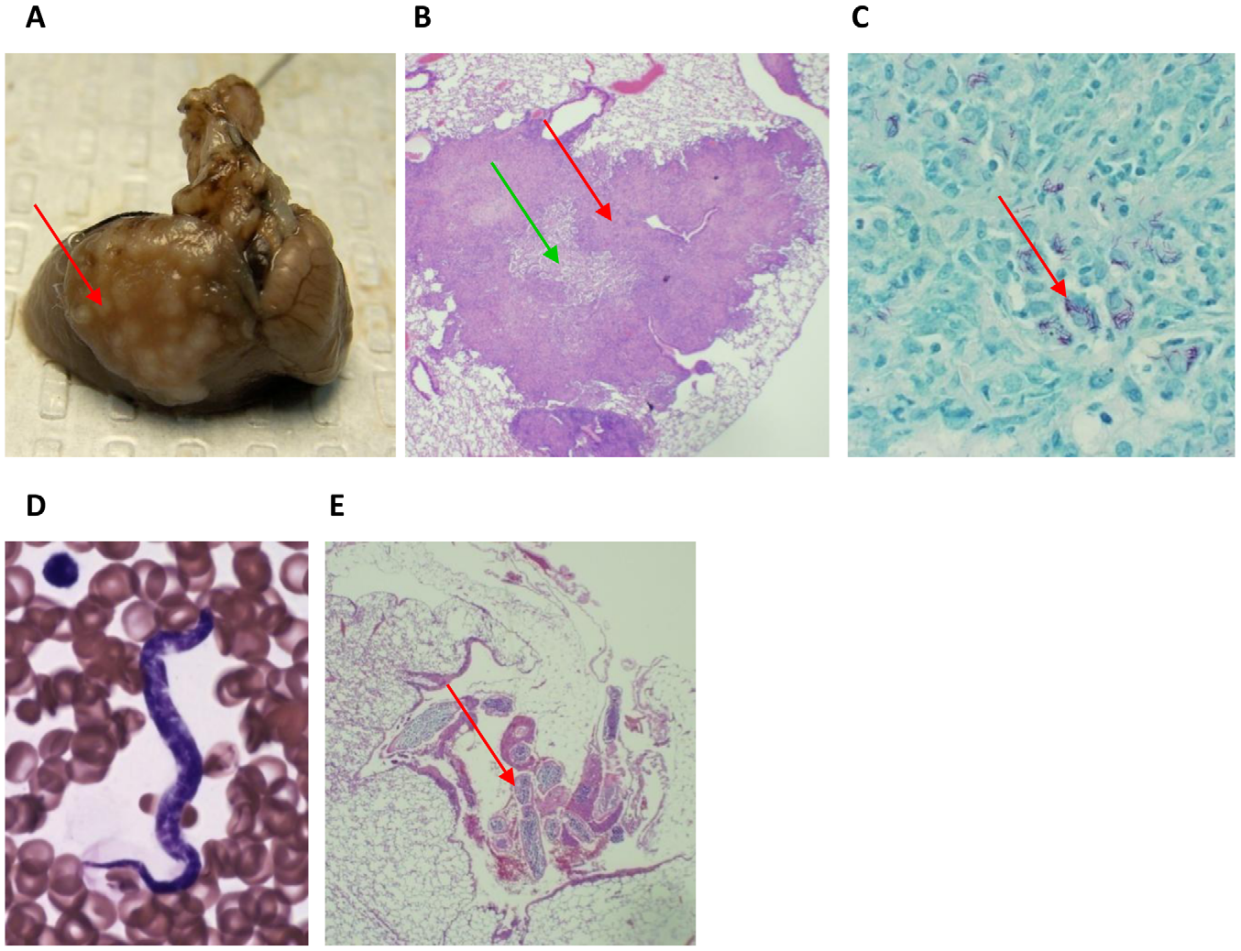
Histological assessment of *L. sigmodontis* and *M. tuberculosis* infection at the 20 week timepoint. *A*, lung with *M. tuberculosis* (MTB) granulomas obtained from a co-infected animal 9 weeks post MTB challenge (the 20 week timepoint). *B*, Lung granuloma (red arrow) with central necrosis (green arrow) observed in the lung of a cotton rat infected with MTB (H&E, 40×). *C*; Acid-fast stain of MTB bacteria in the lung (100×). *D*, *L. sigmodontis* microfilaria in peripheral blood (Eosin-Y Azure A Methylene Blue, 100×). *E*, H&E stained cross-section of lung tissue that shows a *L. sigmodontis* adult worm in the pleural space adjacent to the lung (40×).

At study endpoint, histopathology clearly demonstrated successful infection of cotton rats with MTB and *L. sigmodontis* in all animals. 9 weeks after the challenge with MTB, lungs from cotton rats showed macroscopic granuloma formation (Fig. 3A) with central necrosis (Fig. 3B) and presence of acid-fast stained bacteria (Fig. 3C). Infection with *L. sigmodontis* was confirmed by the occurrence of microfilariae in the peripheral blood (Fig. 3D) and the presence of adult worms in the pleural space adjacant to the lungs (Fig. 3E).

### *M. tuberculosis* co-infection has no consistent impact on *L. sigmodontis* worm burden

Cotton rats are the natural host for *L. sigmodontis* and develop chronic infections. Infectious *L. sigmodontis* larvae migrate after the infection to the pleural cavity and molt into adult worms around 30 days post infection [15]. Microfilariae, the offspring of adult worms, start to be released 8 weeks post infection and circulate in the blood. Peripheral microfilaria counts obtained immediately before the challenge with MTB, 11 weeks post *L. sigmodontis* infection, revealed similar microfilaria levels between both groups (co-infection group: range 0–380 microfilariae/µl, median 180; *L. sigmodontis*-only group: range 82–394, median 152 p = 0.54, data not shown).

**Figure 4:**
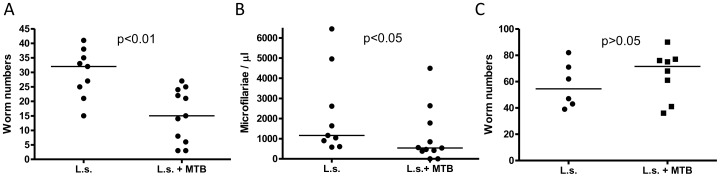
*M. tuberculosis* infection has no consistent impact on *L. sigmodontis* worm burden. *A*, total number of *L. sigmodontis* adult worms recovered from the pleural space and *B*, number of microfilaria per µl of peripheral blood of cotton rats that were infected with *L. sigmodontis* (L.s.) and *M. tuberculosis* (MTB) or *L. sigmodontis* alone (20 weeks post *L. sigmodontis* infection) from the first experiment. *C*, total number of *L. sigmodontis* adult worms recovered from the pleural space of the repeat experiment. Statistical significance was analyzed by the Mann-Whitney-U-test.

The first time we conducted the experiment co-infected cotton rats had significantly fewer microfilariae and adult worms at study endpoint than those in the helminth-only group (median co-infected: 15 adult worms (range 3–27), 540 microfilariae/µl (range 0–4492) vs. median single infected: 32 adult worms (range 15–41), 1163 microfilariae/µl (range 580–6450), Fig. 4A,B). The two cotton rats that did not produce detectable microfilaraemia at study endpoint each had three living adult worms at the end of the experiment and thus were included in the analysis.

However, the reduced adult worm and microfilaria burden in co-infected cotton rats did not occur in the repeat experiment (median co-infected: 71.5 adult worms (range 36–90), 1288 microfilariae/µl (763–3090) vs. median single infected: 54.5 adult worms (39–82), 1416 microfilariae/µl (1287–2146), Fig. 4C, microfilaria counts not shown). These results suggest that MTB co-infection does not have a consistent impact on burden of *L. sigmodontis* infection in cotton rats.

### MTB-only and helminth co-infected cotton rats produce similar amounts of IFNγ

**Figure 5:**
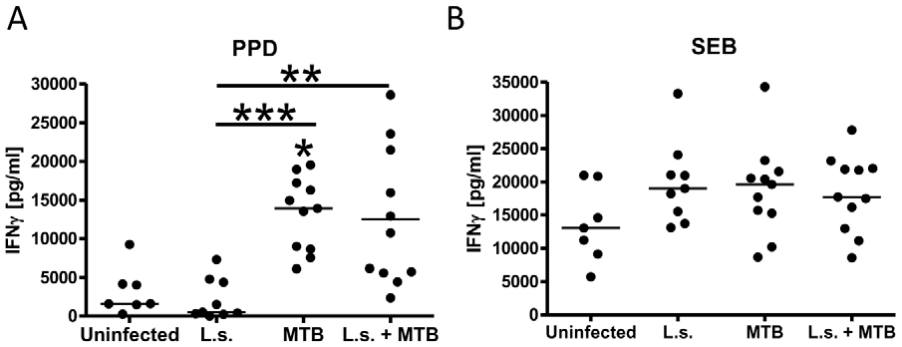
PPD-specific IFNγ production is not reduced by *L. sigmodontis* co-infection. *A*, IFNγ production of spleen cells in response to *M. tuberculosis* PPD and (*B*), SEB. Cotton rats were infected with *L. sigmodontis* (L.s.) and/or *M. tuberculosis* (MTB), or were uninfected. Statistical significance between groups was analyzed by the Kruskal-Wallis test, followed by Dunn's post-hoc multiple comparisons. Shown are representative results from one of two experiments. Single stars show significant differences compared to the uninfected animals. **p<0.01, ***p<0.001.

As IFNγ-driven Th1 immune responses are considered necessary for protection against MTB infection, we tested whether spleen cells from helminth co-infected cotton rats produce less IFNγ in response to PPD compared to cells from MTB-infected controls. In vitro stimulation of splenocytes demonstrated that both MTB-challenged groups produced significantly more IFNγ in response to PPD than uninfected and *L. sigmodontis*-only infected animals (IFNγ in pg/ml: uninfected median 1610 (range 265–9230), *L. sigmodontis*-only 520 (0–7284), MTB-only 13902 (6106–19540), co-infected 10745 (2356–28580), Fig. 5A). Importantly, IFNγ production in co-infected animals was not different than that of cotton rats infected with MTB-only and did not correlate with adult worm (r = −0.182) or microfilaria burden at study endpoint (r = 0.027). Similarly, in the repeat experiment co-infected cotton rats exhibited no reduced PPD-specific IFNγ production from splenocytes compared to MTB-only infected animals, though for all groups levels of PPD-specific IFNγ were lower in the 2^nd^ experiment (data not shown). The total capacity to produce IFNγ from spleen cells was not changed 20 weeks post *L. sigmodontis* infection or 9 weeks after MTB infection, as all groups studied showed similar levels of IFNγ in response to SEB (IFNγ in pg/ml: uninfected median 13060 (range 5740–20990), *L. sigmodontis*-only 19000 (13120–33280), MTB-only 19600 (8640–34280), co-infected 17720 (8560–27800), Fig. 5B). Adult worm numbers tended to be negatively correlated with SEB-induced IFNγ levels (r = −0.5) whereas microfilariae levels had no clear impact (r = −0.22). SEB-induced IFNγ release from splenocytes of uninfected, *L. sigmodontis*-only, and co-infected animals were also similar in the repeat experiment, though MTB-only challenged cotton rats had significantly reduced IFNγ levels compared to uninfected controls (IFNγ in pg/ml: uninfected median 7920 (range 1430–10451), *L. sigmodontis*-only 6274 (2720–7930), MTB-only 1479 (0–5278), co-infected 4383 (1643–7587), data not shown).

### PPD-specific proliferation after MTB challenge is not reduced by *L. sigmodontis* co-infection

**Figure 6:**
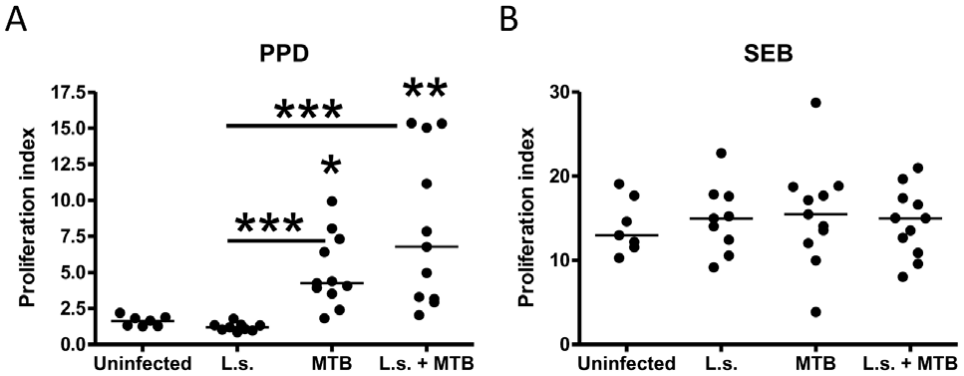
PPD-specific proliferation is not impaired by *L. sigmodontis* co-infection. *A*, Spleen cell proliferation in response to *M. tuberculosis* PPD and *B*, SEB. Cotton rats were infected with *L. sigmodontis* (L.s.) and/or *M. tuberculosis* (MTB), or were uninfected. Shown is the proliferation index (OD of stimulated cells/baseline). Statistical significance between groups were analyzed by the Kruskal-Wallis test, followed by Dunn's post-hoc multiple comparisons. Single stars show significant differences compared to the uninfected animals. Shown are representative results from one of two experiments. **p<0.01, ***p<0.001.

Although chronic helminth infections induce a suppressive, hyporesponsive milieu in their hosts that reduces antigen-specific cell proliferation and can affect the immune response to bystander antigens, helminth co-infection did not reduce PPD-specific spleen cell proliferation during active infection with MTB. Spleen cells from co-infected cotton rats proliferated at least as well as splenocytes from MTB-only challenged animals in response to PPD, and both groups showed significantly increased proliferation rates compared to uninfected or *L. sigmodontis*-only infected animals (proliferation as stimulation index (OD of stimulated cells/baseline): uninfected median 1.65 (range 1.27–2.20), *L. sigmodontis*-only 1.19 (0.85–1.79), MTB-only 4.25 (1.82–9.92), co-infected 6.77 (2.04–15.34), Fig. 6A). PPD-induced spleen cell proliferation indices in the repeat experiment were low and not significantly increased in MTB-only challenged or co-infected cotton rats compared to uninfected or *L. sigmodontis*-only infected animals (uninfected median 1.23 (range 0.64–2.29), *L. sigmodontis*-only 1.00 (0.64–1.50), MTB-only 1.30 (0.81–2.86), co-infected 1.04 (0.80–2.69), data not shown).

Spontaneous and SEB-induced spleen cell proliferation were not affected by *L. sigmodontis* or MTB infection in the first experiment and showed similar results among the different treatment groups (proliferation as stimulation index: uninfected median 12.98 (range 10.28–19.07), *L. sigmodontis*-only 14.99 (9.16–22.74), MTB-only 15.46 (3.86–28.73), co-infected 15.01 (8.03–20.98), Fig. 6B). In the 2^nd^ experiment, while SEB-induced spleen cell proliferation was not significantly different between the various groups, SEB-induced spleen cell proliferation was lowest in *L. sigmodontis*-only infected animals (uninfected median 6.05 (range 1.03–10.51), *L. sigmodontis*-only 2.50 (1.35–9.89), MTB-only 3.63 (0.88–10.66), co-infected 5.42 (3.18–7.81), data not shown).

Total spleen cell numbers were increased in the *L. sigmodontis-*only (median 89.5×10^6^, range 56–110×10^6^), MTB-only (median 105×10^6^, range 42–130×10^6^), and co-infected group (median 83.5×10^6^, range 66–110×10^6^) compared to uninfected controls (median 45×10^6^, range 13–85×10^6^), although this difference was only statistically significant for MTB-only infected animals (p<0.01, data not shown).

### *L. sigmodontis* co-infection does not increase *M. tuberculosis* burden in lung or spleen and does not alter lung granuloma formation

**Figure 7:**
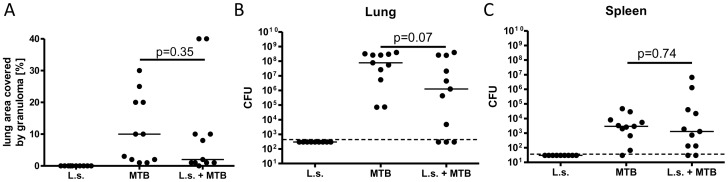
*L. sigmodontis* co-infection does not increase *M. tuberculosis* burden in lung or spleen and does not exacerbate lung granuloma formation. *A*, Percentage of lung area covered with granulomas. *B*, *M. tuberculosis* colony-forming units (CFU) in lung and *C*, spleen. Cotton rats were infected with *L. sigmodontis* (L.s.) and/or *M. tuberculosis* (MTB), or were uninfected. Shown are representative results from one of two experiments. Statistical significance between co-infected and MTB-only infected groups was analyzed by the Mann-Whitney-U-test.

To assess whether helminth infection increases susceptibility to primary MTB infection, lungs from co-infected and MTB-only infected cotton rats were analyzed for granuloma formation and quantitative MTB cultures were conducted on lung and spleen tissues. Helminth co-infection was not associated with greater granulomatous inflammation in the lung compared to MTB-only infected animals (median granuloma area as a percentage of total lung tissue in co-infected animals = 2%, range 0–40% vs. 10% for MTB-only, 1–30%, Fig. 7A). One co-infected animal did not develop lung granulomas, whereas all MTB-only infected animals had lung granulomas. Comparable results were obtained during the repeat experiment, though in general the granuloma-covered area was higher in the repeat experiment (co-infected: median 40%, range 15–75%; MTB-only: median 40%, range 30–75%, data not shown).

Similar to granuloma formation, *L. sigmodontis* co-infection did not increase MTB bacterial burdens in lungs. CFUs from *L. sigmodontis* co-infected and MTB-only infected cotton rats were not significantly different in lung (Fig. 7B), but tended to be lower in the co-infected animals (median 1.2×10^6^ in co-infected vs. 7.8×10^7^ in MTB-only). There was no correlation between adult worm burdens (r = 0.103, 20 weeks post *L. sigmodontis* infection) or microfilaria levels (11 weeks post *L. sigmodontis* infection: r = 0.057; 20 weeks post *L. sigmodontis* r = 0.299) and MTB CFUs in the lungs. Similarly, lung CFUs from co-infected animals tended to be reduced in the repeat experiment compared to MTB-only infected cotton rats (median 3.25×10^7^, range 1.96×10^6^–6.33×10^8^ vs. 2.06×10^8^, range 1.53×10^7^–6.91×10^8^, data not shown). Combined results from both experiments resulted in significantly reduced CFUs in lungs of co-infected animals compared to MTB-only infected cotton rats (p = 0.027). Whereas all animals challenged with MTB-only had positive lung cultures, three co-infected animals in the first experiment had no detectable CFUs in the lung, though two of them had lung granulomas and positive spleen cultures. The co-infected animal without lung granulomas and negative spleen and lung cultures showed the strongest PPD-specific IFNγ production and cell proliferation, suggesting there had been an initial MTB infection which had been successfully cleared. CFUs in the spleen were quantified in the first experiment and were 3–4 logs lower than the ones observed in the lung. Spleen CFUs were similar between helminth co-infected and MTB-only infected cotton rats (median 1300 vs. 2900, Fig. 7C). Similar to lung CFUs, there was no correlation between adult worm burdens (r = 0.048, 20 weeks post *L. sigmodontis* infection) or microfilaria levels (11 weeks post *L. sigmodontis* infection: r = 0.062; 20 weeks post *L. sigmodontis* r = 0.002) and CFUs in spleen. Quantitative MTB cultures from spleens of 2/11 co-infected and 1/11 MTB-only infected animals were negative.

## Discussion

In contrast to the hypothesis that chronic helminth infections worsen the course of MTB, the results of this study demonstrate that chronic filarial infection does not alter control of MTB in the cotton rat. Histological examinations and quantitative MTB cultures from two independent experiments clearly demonstrated equivalent or reduced mycobacterial burden in co-infected animals compared to those infected with only MTB. These findings are supported by immunological studies revealing that PPD-specific cellular proliferation and IFNγ production were not suppressed in co-infected animals.

These results are unexpected since it is documented that chronic helminth infection can alter the immune response to bystander antigens. Indeed, recent studies have shown that chronic filarial infection is associated with decreased PPD-specific IFNγ and IL-17 responses in individuals latently infected with tuberculosis [18]. Similarly, active filaria infection in patients latently infected with MTB correlates with a reduction in TLR2 and TLR9 activation in response to MTB antigens that normalizes after anti-filarial treatment [19].

The results of our study, however, suggest that systemic filaria-induced immunomodulation can be overcome in the setting of an active MTB infection. Immunomodulatory effects in cotton rats chronically infected with *L. sigmodontis* were confirmed in our model. A time course study showed that cotton rats infected with *L. sigmodontis* developed eosinophilia, which correlates with the induction of a Type 2 immune response. Additionally, splenocytes of cotton rats infected for 11 weeks exhibited reductions in parasite-specific cytokine production and splenocyte proliferation in response to polyclonal activation.

Among previous in-vivo co-infection studies utilizing helminths and mycobacteria, two showed no effect of helminths on mycobacterial infection and three observed worsened control [20], [21], [22], [23], [24]. The first two studies which demonstrated negative impact used an intravenous *Mycobacterium bovis* infection challenge into mice either chronically infected with *Schistosoma mansoni* [23] or acutely infected with *Strongyloides venezuelensis* [20]. A possible explanation for the observed difference between those studies and ours may be that distinct helminths have different effects on mycobacteria co-infection. Filariae, strongylids, and schistosomes all reside in different tissue spaces, have markedly different lifecycles, and release different excretory/secretory factors. For example, it has been shown that in vitro exposure of human dendritic cells to microfilariae of the human filaria *Brugia malayi* results in decreased expression of DC-sign, a receptor for MTB [25], providing a potential mechanism by which filariae may actually have some host-protective effects against tuberculosis.

In addition to the different effects parasites may induce on host cells, the anatomical niche used by helminths inside the host may be important for impacting the immune response to mycobacteria. The helminth utilized in our study lives in close proximity to MTB. *L. sigmodontis* adult worms live in the pleural space abutting the lungs, and microfilariae enter the peripheral blood via the lung capillaries and regularly transit through the spleen. Thus, there is potential for *L. sigmodontis* to exert local effects on MTB co-infection. For example, the influx of cells into the pleural cavity induced by adult *L. sigmodontis* worms could potentially facilitate clearance of MTB bacteria.

Alternatively, it is possible that the differences observed between the *M. bovis* models [20], [23] and ours were due to the different mycobacterial models used. One of the strengths of our study was the use of the cotton rat model of MTB infection. Unlike murine mycobacterial models, MTB infection of cotton rats results in discrete granulomas containing macrophages, mycobacteria, and central necrosis similar to that observed in human tuberculosis. MTB and schistosome co-infection in the cotton rat may reveal whether individual helminths have different effects on the course of mycobacterial infection.

The third study that has shown a negative impact of helminth infection on control of mycobacteria utilized *Nippostrongylus brasiliensis* infection in mice [21]. In this study, acute infection with 500 tissue-invasive *N. brasiliensis* larvae transiently worsened control of *M. tuberculosis* infection in an acute setting [21]. The contrasting outcomes of this study and ours are likely due to differences in the helminth models as well as the timing of the MTB challenge. Whereas *N. brasiliensis* L3 infections induce a short-lived infection in mice, chronic *L. sigmodontis* infection persists in cotton rats for years. Thus, it can be assumed that *L. sigmodontis* worms are better adapted to the immune system of their natural host, the cotton rat. As such, chronic *L. sigmodontis* infection in cotton rats is likely a good immunologic model for long-term persistent human filarial infections. Another key difference between the *N. brasiliensis* model and ours is the timing of MTB infection. Whereas MTB challenge was given only days after *N. brasiliensis* infection, when type 2 immune responses are increasing, we challenged rats with MTB 11 weeks after helminth infection, a timepoint at which chronic helminth infection and immunoregulatory responses have become established. Whether acute *L. sigmodontis* infection imparts a transient reduction in control of MTB infection similar to *N. brasiliensis* is not known and may be the topic of future studies.

In addition to the in vivo animal studies that showed a negative impact of helminths on mycobacterial infection, Elias et al. showed that acute MTB infected patients had an increased frequency of helminth infection compared to MTB negative household contacts [26]. This discrepancy with our results may also be due to differences in the helminth species present in the hosts. While in our experiments a filarial nematode was used, Elias et al. observed an increased frequency of helminth infection with Schistosomes and intestinal nematodes (hookworms, *Ascaris*, *Trichuris*, *Strongyloides*). In contrast, a different epidemiological study done in South India found no impact of either intestinal or filarial infection on frequencies of PPD positivity [27].

It is important to note that our study did not evaluate the effects helminth infections have on latent MTB. As the immune response required for control of latency may be different than that required for control of active disease, it may be worthwhile exploring whether chronic helminth infection alters the risk of reactivation in a latent MTB model.

Interestingly, in the first co-infection experiment we conducted adult *L. sigmodontis* worm numbers and microfilaria counts were significantly decreased in MTB co-infected cotton rats. While Th2 immune responses are generally considered protective against helminth infections, we speculate that the decreased worm burden was due to the pro-inflammatory environment created by the MTB co-infection, as it is known that IFNγ can contribute to resistance against *L. sigmodontis* [28]. In accordance with this speculation, IFNγ production from splenocytes of all groups were lower in the second experiment, correlated with a higher worm burden 20 weeks post *L. sigmodontis* infection, and was associated with no difference in worm burdens of co-infected and single infected groups.

In conclusion, our data demonstrates that chronic filaria infection does not exacerbate the course of acute MTB in the cotton rat model. While results of prior studies investigating the effects helminth infections have on MTB co-infection have been conflicting, we believe that the use of an animal in which the host develops granulomas to MTB in combination with a chronic helminth infection in its natural host makes this study the most likely to approximate chronic helminth infection and MTB co-infection in humans.

While our results indicate that filaria eradication programs may not have a substantial impact on MTB control, they also suggest that it may be possible to develop worm-derived therapies for autoimmune diseases which do not substantially increase the risk for severe infections. Future studies evaluating effects of different helminths utilizing the same MTB model and assessing the impact of helminths in MTB latency models will provide important insights for further understanding the effects helminth co-infections have on MTB.
